# Is immunotherapy an effective treatment for Alzheimer's disease?

**DOI:** 10.1186/1742-4933-1-3

**Published:** 2004-11-12

**Authors:** Federico Licastro, Calogero Caruso

**Affiliations:** 1 Dipartimento di Patologia Sperimentale, Università di Bologna, Via S. Giacomo 14, 40126 Bologna, Italy; 2 Gruppo di Studio sull'Immunosenescenza, Dipartimento di Biopatologia e Metodologie Biomediche, Università di Palermo, Corso Tukory 211, 90134 Palermo, Italy

## Abstract

Immunotherapy in patients with Alzheimer's disease (AD) is rapidly becoming a hot topic of modern geriatric and clinical gerontology. Current views see immunization with Aβ peptide, the amyloidogenic protein found in senile plaque of AD patient's brains, or the infusion of preformed antibody specific for human Aβ, as possible therapeutic approaches to improve the cognitive status in the disease. Animal models of the disease have provided positive results from both approaches. Thus, an initial clinical trial using immunization with human Aβ in AD patients was started, but then shortly halted because of an unusually high incidence (6%) of meningoencephalitis. A long and currently ongoing debate in the scientific community about the pro or contra of vaccination or passive immunization with Aβ in AD is thereafter started. Here, the authors would like to stress few points of concern regarding these approaches in clinical practice.

Immunotherapy in patients with Alzheimer's disease (AD) is rapidly becoming a hot topic of modern geriatric and clinical gerontology. M.E. Weksler [1] in the article entitled "The immunotherapy of Alzheimer's disease" published in Immunity and Ageing discussed this theme.

Current views see immunization with Aβ peptide, the amyloidogenic protein found in senile plaque of AD patient's brains, or the infusion of preformed antibody specific for human Aβ, as possible therapeutic approaches to improve the cognitive status in the disease.

Animal models of the disease have provided positive results from both approaches, since either vaccination with human Aβ or infusion of preformed antibodies specific for Aβ, have resulted in a decrease of amyloid plaques density in the brain of amyloid precursor protein (APP) transgenic mice [2,3]. Improved memory performances after Aβ vaccine on APP transgenic mice have also been claimed [4]. Several other studies from transgenic mice have there after reinforced the suggestion that vaccination or passive immunotherapy might result in a relevant clinical effect in human patients (see the Weksler article [1]).

An initial clinical trial using immunization with human Aβ in AD patients was started and then shortly halted because of an unusually high incidence (6%) of meningoencephalitis (see the Weksler article [1]). A long and currently ongoing debate in the scientific community about the pro or contra of vaccination or passive immunization with Aβ in AD is thereafter started.

Here, we would like to stress few points of concern regarding these approaches in clinical practice.

1) The claimed animal model for AD is unfortunately incomplete even if useful model for the human disease.

2) Vaccination with human Aβ in mice induces an immune response against a foreign protein, i.e. human Aβ, and the mouse Aβ homolog does not appear to be involved. On the contrary, Aβ vaccination in man may potentially induce an autoimmune like disease in the brain and other peripheral tissues of susceptible patients. At the moment we do not have the ability to predict which patients will suffer destructive immune responses.

3) The effects of both vaccination or passive immune therapy in AD brains might be non-specific, as already suggested by a recent report [5] and by the original histopathological investigation from AD patients deceased after meningoencephalitis [6]. This notion was then reinforced by the report of these authors [7].

4) Vaccine therapy is not always effective in the elderly, since immune defects of variable degree are often present in old persons [8]. In old clinically ill AD patients this immune activation might fail or activate noxious auto-aggressive immune responses. Once again we cannot predict which patient will experience one or the other condition after the vaccination.

5) Historically vaccination has been successful in the prevention of the diseases much and much less in the therapy of ongoing diseases. A significant gain would be achieved by Aβ vaccination or passive immune therapy, if these manipulations will work in the very early stages of the disease. At the moment, no clinical data on this topic are available and those from animal models have not extensively addressed this topic.

6) A general consideration on AD is also mandatory. In fact, clinical signs of dementia show up after extensive synapse and neuron loss have already occurred in the brain. How can an immune response restore an already compromised nervous circuit or revive dead neurons? The very modest decrement of cognitive deterioration rate claimed in a proportion of AD patients receiving the vaccine is a marginal therapeutic goal. In fact, the disease has not been cured, and the modest clinical slow down in the AD progression takes big prices, i.e. patients and care givers will continue to suffer the catastrophic effects of the disease for a longer time.

We feel that Aβ vaccination and in a less extent passive immune therapy are now exciting experimental protocols for animal research and experimental neurology investigations, probably premature for clinical applications.
